# Chloroquine efficacy for *Plasmodium vivax* in Myanmar in populations with high genetic diversity and moderate parasite gene flow

**DOI:** 10.1186/s12936-017-1912-y

**Published:** 2017-07-10

**Authors:** Myo Win Htun, Nan Cho Nwe Mon, Khin Myo Aye, Chan Myae Hlaing, Myat Phone Kyaw, Irene Handayuni, Hidayat Trimarsanto, Dorina Bustos, Pascal Ringwald, Ric N. Price, Sarah Auburn, Kamala Thriemer

**Affiliations:** 1 Department of Medical Research, Yangon, 11191 Myanmar; 2 Global and Tropical Health Division, Menzies School of Health Research and Charles Darwin University, Darwin, NT 0810 Australia; 3 Eijkman Institute for Molecular Biology, Jl. Diponegoro 69, Central Jakarta, 10430 Indonesia; 4 The Ministry of Research and Technology (RISTEK), Jakarta, Indonesia; 5 Agency for Assessment and Application of Technology, Jl. MH Thamrin 8, Jakarta, 10340 Indonesia; 6 World Health Organization, Country Office for Thailand, Ministry of Public Health, Nonthaburi, Thailand; 7 Global Malaria Programme, World Health Organization, 20 Avenue Appia, 1211 Geneva, 27, Switzerland; 8 Centre for Tropical Medicine and Global Health, Nuffield Department of Medicine Research Building, University of Oxford Old Road Campus, Oxford, UK

**Keywords:** Malaria, Plasmodium, Vivax, Chloroquine, Efficacy, Resistance, Genotyping, Diversity, Transmission, pvmdr1, Copy number, Myanmar

## Abstract

**Background:**

*Plasmodium vivax* malaria remains a major public health burden in Myanmar. Resistance to chloroquine (CQ), the first-line treatment for *P. vivax*, has been reported in the country and has potential to undermine local control efforts.

**Methods:**

Patients over 6 years of age with uncomplicated *P. vivax* mono-infection were enrolled into clinical efficacy studies in Myawaddy in 2014 and Kawthoung in 2012. Study participants received a standard dose of CQ (25 mg/kg over 3 days) followed by weekly review until day 28. *Pvmdr1* copy number (CN) and microsatellite diversity were assessed on samples from the patients enrolled in the clinical study and additional cross-sectional surveys undertaken in Myawaddy and Shwegyin in 2012.

**Results:**

A total of 85 patients were enrolled in the CQ clinical studies, 25 in Myawaddy and 60 in Kawthoung. One patient in Myawaddy (1.2%) had an early treatment failure and two patients (2.3%) in Kawthoung presented with late treatment failures on day 28. The day 28 efficacy was 92.0% (95% CI 71.6–97.9) in Myawaddy and 98.3% (95% CI 88.7–99.8) in Kawthoung. By day 2, 92.2% (23/25) in Myawaddy and 85.0% (51/60) in Kawthoung were aparasitaemic. Genotyping and *pvmdr1* CN assessment was undertaken on 43, 52 and 46 clinical isolates from Myawaddy, Kawthoung and Shwegyin respectively. *Pvmdr1* amplification was observed in 3.2% (1/31) of isolates in Myawaddy, 0% (0/49) in Kawthoung and 2.5% (1/40) in Shwegyin. Diversity was high in all sites (*H*_E_ 0.855–0.876), with low inter-population differentiation (*F*_ST_ 0.016–0.026, *P* < 0.05).

**Conclusions:**

Treatment failures after chloroquine were observed following chloroquine monotherapy, with *pvmdr1* amplification present in both Myawaddy and Shwegyin. The results emphasize the importance of ongoing *P. vivax* drug resistance surveillance in Myanmar, particularly given the potential connectivity between parasite population at different sites.

**Electronic supplementary material:**

The online version of this article (doi:10.1186/s12936-017-1912-y) contains supplementary material, which is available to authorized users.

## Background

Malaria remains a major public health burden in Myanmar, with an estimated 240,000 cases in 2015 of which 78,000 were confirmed [1]. The control and elimination of malaria in Myanmar is critical for reducing the national burden as well as that for eliminating the disease in neighbouring countries, which account for approximately 75% of all reported cases in the Greater Mekong Sub-region [1]. The transmission of malaria across borders via mobile and migrant populations is a major challenge in the region [2, 3]. The threat of multidrug resistant *Plasmodium falciparum* has prompted the National Malaria Control Programme (NMCP) to set a goal to eliminate indigenous malaria in Myanmar by 2030. Containing the spread of artemisinin resistant *P. falciparum* is the clear priority, but *P. vivax* also presents a considerable challenge to local malaria elimination. Whilst *P. vivax* currently accounts for only a third of all malaria cases in Myanmar, the NMCP anticipates that eliminating this species will take considerably longer than for *P. falciparum*. Indeed, the proportion of malaria cases caused by *P. vivax* infection in this region has increased steadily since 2012 [1]. Several biological features of *P. vivax* render this species highly resilient to transmission intervention and afford high resurgence potential including the low, often sub-microscopic density of infection, the early development of transmissible stages (gametocytes) before the patient becomes clinically unwell, and the propensity to form dormant liver stages (hypnozoites) that may persist for months to years before being reactivated [4].

The NMCP recommends a combination of chloroquine (CQ) and primaquine as first-line therapy for radical cure of *P. vivax* malaria. However, reports of declining efficacy across increasing proportions of the vivax-endemic world [5], including neighbouring Thailand [6], and sporadic reports of treatment failure within the country [7–9], demands diligent surveillance of CQR *P. vivax* in Myanmar. Relative to clinical and ex vivo surveys, molecular methods offer a simple and cost-effective strategy for the wide scale surveillance of antimalarial drug resistance. However, whilst mutations in orthologues of chloroquine resistance (CQR) determinants in *P. falciparum* including *pvcrt*-*o* (*P. vivax* chloroquine resistance transporter-O) and *pvmdr1* (*P. vivax* multidrug resistance transporter) have been evaluated in Myanmar [10], these markers have not been validated as CQR determinants for *P. vivax* [11].

In countries where *P. falciparum* and *P. vivax* are co-endemic therapies used to treat *P. falciparum* have also been shown to induce reduced efficacy in *P. vivax*, presumably through incidental exposure [12]. A recent molecular study of *P. vivax* in the Thai–Myanmar border region revealed a common copy number (CN) amplification of *pvmdr1* associated with mefloquine (MQ) resistance [13, 14], that appeared to have arisen under drug pressure from artesunate–mefloquine (ASMQ) regimens used to treat the co-endemic *P. falciparum* population [15]. In Myanmar, the NMCP recommends artemether–lumefantrine (AL) as first-line treatment of *P. falciparum* malaria, but treatment failure occurs within 28 days, other artemisinin-based combinations, such as ASMQ or dihydroartemisinin–piperaquine (DHP) are advised. Whilst molecular markers of *P. vivax* susceptibility to artemisinin and piperaquine remain unknown *pvmdr1* amplification has been associated with MQ resistance [13]. More recently, a simple breakpoint PCR method has been used to detect pvmdr1 amplification in Thai isolates [15], but has not yet been assessed in other populations.

Amidst the threat of antimalarial drug resistance emergence, the NMCP is striving to control and eliminate *P. vivax* malaria as rapidly as possible with the available resources, requiring effective characterization of local transmission intensity and stability. Whilst measures of parasite prevalence or incidence are helpful to gauge transmission intensity, these methods can underestimate the burden of malaria, particularly of *P. vivax*, where infections are often sub-patent and/or asymptomatic [16]. Genetic analysis of the parasite is a complementary approach to strengthen local estimates of transmission intensity and stability through characterization of parasite diversity and relatedness, as well as offering an approach to gauge parasite gene flow between different populations, and the associated risks of drug resistance spread [17, 18].

To better understand the local dynamics of *P. vivax* anti-malarial drug resistance, and the heterogeneity in transmission intensity and stability, clinical and molecular surveys at three sites in the south-eastern region of Myanmar were conducted. The efficacy of chloroquine against *P. vivax* was assessed at two sites using the standard WHO 28-day clinical efficacy protocol. Molecular surveillance including microsatellite genotyping and *pvmdr1* CN was undertaken in all three sites to assess local patterns in parasite diversity and associated transmission dynamics and to gauge the selective pressure of MQ regimens on the local *P. vivax* population.

## Methods

### Study sites and participants

**Fig. 1:**
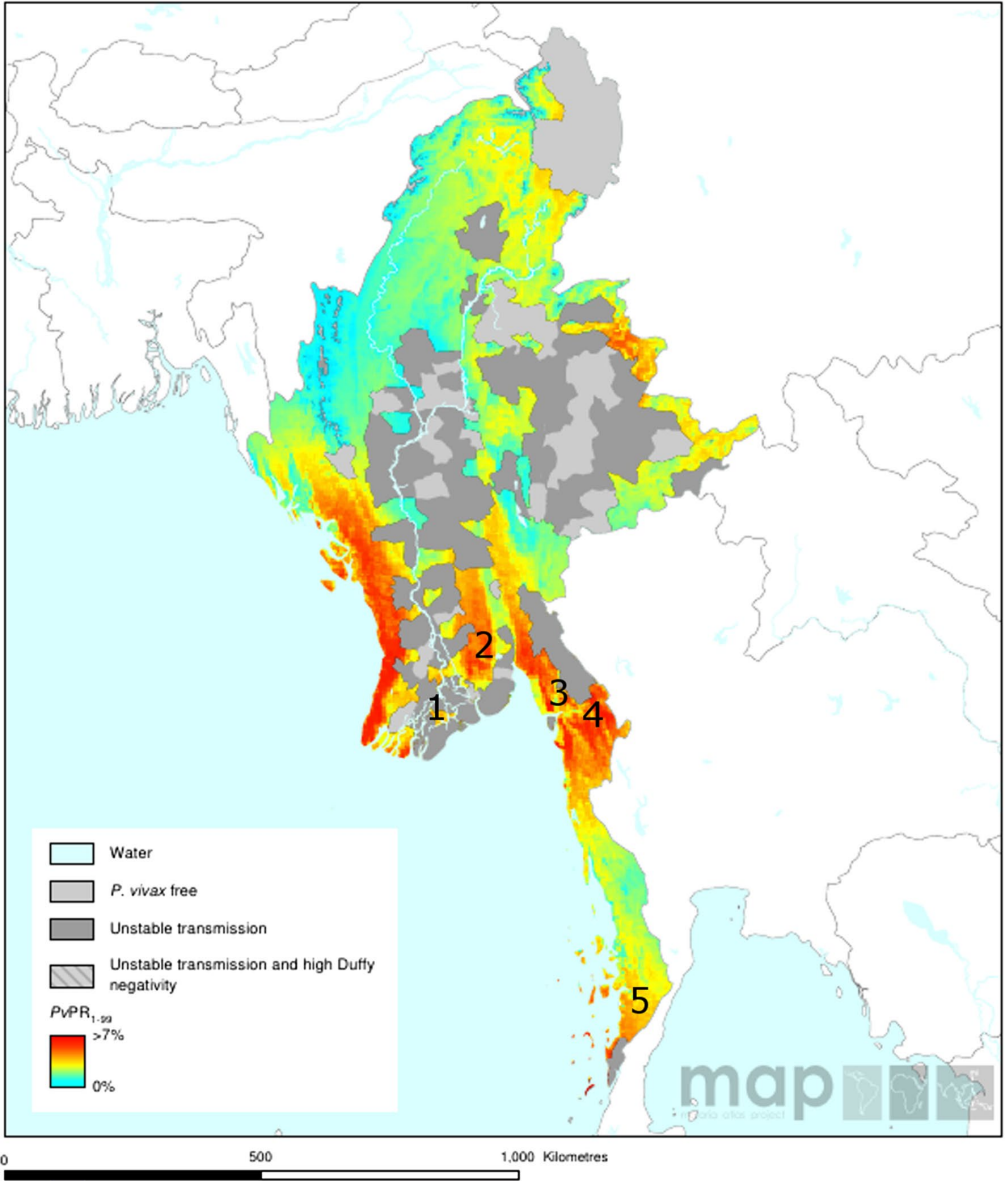
*Plasmodium vivax* prevalence map illustrating study site locations. This map was generated by the Malaria Atlas Project, University of Oxford. The *colour scales* reflect the model-based geostatistical point estimates of the annual mean *P. vivax* parasite rate in the 1–99 year age range (*Pv*PR_1–99_) [54] within the stable spatial limits of transmission in 2010. The approximate locations of the study sites described here are indicated with numbers as follows; *1* Insein, Yangon Region; *2* Shwegyin, Bago East Region; *3* Hpa-An, Kayin State; *4* Myawaddy, Kayin State; *5* Kawthoung, Tanintharyi Region

A map illustrating the location of the study sites is presented in Fig. 1, and additional details on the study sites are provided in Additional file 1. The CQ efficacy studies were conducted at two sites: Myawaddy (Kayin State) in 2014 and Kawthoung (Tanintharyi Region) in 2012. Molecular analysis was undertaken on the samples from the CQ efficacy studies as well as additional samples collected from symptomatic patients presenting to clinics in Myawaddy in 2012, Insein, Yangon Region in 2014, Shwegyin, Bago East Region in 2012–13, and Hpa-An, Kayin State in 2014.

In 2012, *P. vivax* malaria in Kayin State, Tanintharyi Region and Bago East Region was defined as moderate stable endemic or ‘hypo-mesoendemic’ [19]. According to the API levels in 2012 (all greater than 1 case per 1000 population at risk), Myawaddy, Kawthoung and Shwegyin townships are classified as category 1 settings and areas that are still in the transmission reduction phase where elimination of malaria does not appear to be feasible at present.

### Clinical study protocol

#### Study population

Patients attending one of the two health centres were screened for inclusion into the study. Inclusion criteria were as follows: confirmed mono-infection with *P. vivax* with a parasite density ≥250 asexual parasites per microliter (µl), age >6 years, axillary temperature ≥37.5 °C or history of fever present within previous 24 h, ability to swallow oral medication, ability and willingness to comply with the study protocol for the duration of the study and to comply with the study visit schedule. Written informed consent from the patient or in case of minors from a parent or guardian was obtained. Exclusion criteria were presence of general danger signs or signs of severe malaria, mixed infections, severe malnutrition, underlying chronic or severe disease, and history of hypersensitivity to the study drug. Pregnant or lactating women were excluded as well as women of childbearing age between 12 and 18 years because of the risk of unexpected pregnancy.

#### Study procedures

The study used the WHO 28-day protocol to assess efficacy of chloroquine against *P. vivax*. Individuals with uncomplicated vivax malaria, who met the inclusion criteria were enrolled. Chloroquine was given at 25 mg base/kg body weight divided over 3 days (10 mg base/kg on day 0 and 1 and 5 mg base/kg on day 2). Study participants were observed for 30 min after treatment for adverse reactions or vomiting. Patients who vomited their first dose were treated again with the same dose of chloroquine and were observed for an additional 30 min. Primaquine treatment was delayed until the end of the follow up.

After completion of the treatment course patients were asked to return to the enrolment centre on day 3 and then for weekly follow up visits on day 7, 14, 21 and 28 and whenever signs and symptoms consistent with malaria were present. Patients who didn’t come back for their scheduled visits were contacted and asked to return at the earliest convenience. A general physical exam was done during the enrolment visit. Baseline data on age, sex, weight and height was collected. Adverse events were recorded at every visit. A finger prick blood sample was taken at every follow up visit and any unscheduled visit for microscopy.

#### Study endpoints

The primary study endpoint was the response to treatment on day 28. Patients were divided into treatment failures (early or late failures) and patients with adequate clinical and parasitological response according to WHO criteria [20]. In brief: early failures were defined as parasitaemia on day 2 higher than on day 0 irrespective of axillary temperature, parasitaemia on day 3 with axillary temperature ≥37.5 °C or parasitaemia on day 3 ≥25% of count on day 0. Late failures were defined the presence of parasitaemia on any day between day 4 and day 28 in patients who did not previously meet any of the criteria of early treatment failure. Patients with no parasitaemia on day 28 and who did not previously meet any of the criteria of treatment failure were defined as adequate clinical and parasitological response (ACPR). Parasite clearance was presented as the proportion of patients with microscopy negative results within the first 3 days.

#### Microscopy

Thick and thin blood films were prepared at each visit. Giemsa-stained thick and thin blood films were examined. Parasite density, expressed as the number of asexual parasites per µl of blood, was calculated by dividing the number of asexual parasites by 200 WBCs counted and then multiplying by an assumed WBC density of 6000 WBCs/µl. A blood slide was considered negative when the examination of 1000 WBCs did not reveal any asexual parasites. All slides were read by two qualified microscopists independently. Parasite densities were calculated by averaging the two counts. Blood smears with discordant results (differences between the two microscopists in species diagnosis, or differences in parasite density of >50% or difference in the presence of parasites) were re-examined by a third, independent microscopist, and parasite density calculated by averaging the two most concordant counts.

#### Statistical analyses

All analyses were performed using STATA 12 (StatCorp, US). Descriptive statistics were computed and differences were compared using either a Chi square test for categorical variables, or the Mann–Whitney U test or the nonparametric equality-of-medians test as required for continuous variables. Treatment success was assessed by Kaplan–Meier analyses.

### Molecular study protocols

#### DNA extraction and species confirmation

DNA was extracted from dried blood spots using the QIAamp DNA Blood mini kit (Qiagen) according to the manufacturer’s instructions for dried blood spots. The presence of *P. vivax* versus other *Plasmodium* species was confirmed by PCR using the protocol described by Padley et al. [21]. for detection of *P. vivax*, *P. falciparum*, *Plasmodium malariae* and *Plasmodium ovale*, and the protocol of Imwong et al. [22] for the detection of *Plasmodium knowlesi*. The Padley assays were modified slightly such that each species was diagnosed in a separate (non-multiplex) assay.

#### STR genotyping

Genotyping was undertaken at nine previously described short tandem repeat (STR) markers that are included in a consensus panel selected by partners within the Vivax Working Group of the Asia Pacific Malaria Elimination Network (APMEN): *Pv3.27, msp1F3, MS1, MS5, MS8, MS10, MS12, MS16* and *MS20* [23, 24]. The loci were amplified using methods described previously [25]. The labelled PCR products were sized by denaturing capillary electrophoresis on an ABI 3100 Genetic Analyzer with GeneScan LIZ-600 (Applied Biosystems) internal size standards. Genotype calling was undertaken using VivaxGEN version 1.0 [26]. An arbitrary fluorescent intensity threshold of 100 relative fluorescence units was applied for peak detection, and minor alleles were only called if they had a minimum 33% height of the predominant allele, a threshold that has been applied in all APMEN and many other *P. vivax* genotyping studies. All electrophoretogram traces were additionally inspected manually.

#### Pvmdr1 copy number breakpoint assay

The prevalence of the *pvmdr1* amplification with conserved breakpoints in Thailand was assessed in the Myanmar isolates using the methods detailed by Auburn et al. [15]. Briefly, the methods comprise three assays including two positive control assays utilizing primer pairs MDR1LF (5′-ACTGCGAAAGTCGCCTATTT-3′) and MDR1LR (5′-TCATCGTGTGGCACATTTTT-3′), and MDR1RF (5′- GGTGAAAAGGTCGAAGCAAA-3′) and MDR1RR (5′-GGGACACGTTCCTCAGAAGT-3′) which should amplify 408 and 505 bp products respectively in all *P. vivax* isolates, and a test assay comprising MDR1RF and MDR1LR, which amplify a ~600 bp product spanning the junction between tandem copies in multi-copy (CN2+) isolates with the ‘Thai’ amplification breakpoint. In isolates without the amplification, MDR1RF and MDR1LR are positioned in opposite orientations and so will not yield a product. Samples were run in duplicate on all three assays, and discordant results were repeated in duplicate. The test assay was only considered successful if both positive control assays amplified the expected products.

#### Population genetic analysis

Polyclonal infections were identified by the presence of two or more alleles at any of the nine markers assessed within a given sample. The multiplicity of infection (MOI) for a given sample was defined as the maximum number of alleles observed at any of the nine markers investigated. To ensure unbiased estimation of the allele frequencies in the population, with the exception of measures of polyclonality and MOI, only the predominant allele at each locus in each isolate was included in analysis [27].

Population-level diversity was assessed using the expected heterozygosity (*H*_E_) measure. *H*_E_ was calculated using the formula [*n*/(*n* − 1)] [1 − Σ*p*_*i*_^2^], where *n* is the number of isolates analysed and *pi* is the frequency of the *i*th allele in the population. In addition to the full spectrum of nine markers, sub-analyses of the *H*_E_ and genetic differentiation (described below) were undertaken on a sub-set of five markers (MS1, MS5, MS10, MS12, MS20) defined as exhibiting balanced diversity in a recent study [28].

The genetic distance between populations was measured using the pairwise *F*_ST_ metric, implemented with Arlequin software (version 3.5) [29]. Standardized measures (*F*_ST_^′^) were also calculated to adjust for potentially high marker diversity [30]. The results were interpreted according to the classification of Balloux et al. as follows; *F*_ST_ < 0.05 = little/no differentiation, 0.05 ≥ *F*_ST_ < 0.15 = moderate differentiation; 0.15 ≥ *F*_ST_ < 0.25 = high differentiation; and *F*_ST_ > 0.25 = very high differentiation [31]. Further assessment of the population structure was undertaken using STRUCTURE software version 2.3.3 [32]. For each of 1–10 K (sub-populations), 20 replicates were run with 100,000 burn-in and 10,000 post burn-in iterations. Model parameters included allowance for admixture, and correlated allele frequencies. The most probable number of sub-populations (*K*) was derived by applying the delta K method [33].

In the isolates with successful allele calls for all nine markers, multi-locus genotypes (MLGs) were reconstructed from the predominant allele at each locus for assessment of the genetic relatedness between sample pairs. Genetic relatedness was measured as the proportion of alleles shared between MLG pairs (*ps*). Using (1 − *ps*) as a measure of genetic distance [34], an unrooted neighbour-joining tree [35] was generated with the ape package in R [36].

The MLGs were also used to assess multi-locus linkage disequilibrium (LD). LD levels were measured using the standardized index of association (*I*_A_^S^) implemented with the web-based LIAN 3.5 software, with significance estimates determined using 10,000 random permutations of the data [37]. For each population, LD was assessed in (1) all samples, (2) samples with a maximum of one multi-allelic locus (i.e. low complexity samples), and (3) with each unique haplotype represented just once (i.e. unique set of MLGs).

## Results

### Clinical efficacy study

#### Study profile and baseline characteristics

A total of 85 patients were enrolled at two sites in Myanmar between August 2012 and October 2014: 60 (70.6%) patients in Kawthoung and 25 (29.4%) in Myawaddy. Adherence to follow up was 100% (25/25) in Myawaddy, and 92.9% (59/65) in Kawthoung. Patients in Myawaddy also had significantly higher parasite densities at enrolment (Table 1).

**Table 1:** Baseline characteristics of patients in the clinical study

	Overall (n = 85)	Myawaddy (n = 25)	Kawthoung (n = 60)	*P* value
Gender male n (%)	61 (71.8%)	22 (88.0%)	39 (65.0%)	0.032
Median age in years	24	22	24.5	0.41
Age classes n (%), years				
>5 to ≤15	20 (23.5%)	6 (24.0%)	14 (23.3%)	0.95
>15	65 (76.5%)	19 (76.0%)	46 (76.7%)	
Median height in cm	154	165	152	0.008
Median weight in kg	57	63	52	0.002
Mean temperature in °C	38.3	38.1	38.4	0.01
Geomean parasite density, µl^−1^	5637	11,002	4267	0.001

#### Efficacy outcomes

**Fig. 2:**
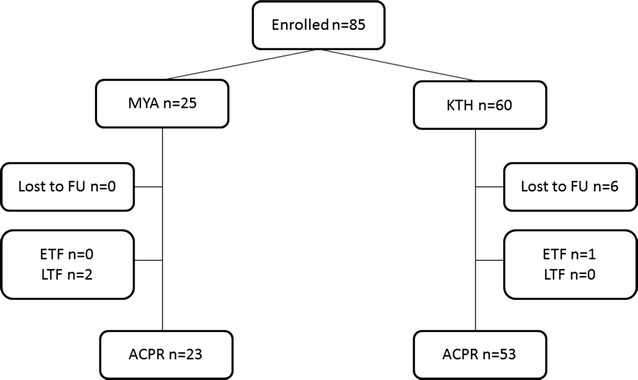
Flowchart of patients in the clinical study. *MYA* Myawaddy, *KTH* Kawthoung

A total of 85 patients were included in the survival analyses, of whom one patient in Kawthoung had an early treatment failure (parasitaemia in day 3 and 37.5 °C body temperature) and two patients in Myawaddy had recurrent *P. vivax* parasitaemia by day 28 (Fig. 2). The day 28 efficacy of chloroquine was 92.0% (95% CI 71.6–97.9) in Myawaddy and 98.3% (95% CI 88.7–99.8) in Kawthoung.

By day 2, 87.1% (74/85) of patients were aparasitaemic with parasite clearance not significantly faster in Myawaddy (92.2%, 23/25) compared to Kawthoung (85.0%, 51/60); P = 0.5. All but the one patient (1.2%, P = 0.2) defined as early treatment failure at the site in Kawthoung had cleared their parasites by day 3. The late treatment failures cleared their parasites on day 2 and 3.

The mean total dose of CQ taken was 26.8 mg/kg (SD = 4.7 mg/kg). Patients with treatment failure received a lower mean dose of chloroquine [22.9 mg/kg (SD = 1.0 mg/kg)] than those without treatment failure [26.9 mg/kg (SD = 4.7 mg/kg)]; P = 0.05. The patient with the early treatment failure had the lowest dose with 21.7 mg/kg. None of the patients vomited their treatment doses or needed re-dosing.

### Molecular studies

#### Molecular processing summary

Molecular processing was undertaken on samples collected within the clinical study, as well as additional samples collected from cross-sectional studies conducted over the same time span (Additional file 2). A total of 152 mono-species *P. vivax* (151/152, 99.3%) or mixed-species *P. vivax* + *P. falciparum* (1/152, 0.7%) infections were confirmed by PCR. Three study sites had sufficient sample size for population-level genetic analyses; Shwegyin (*n* = 46), Myawaddy (*n* = 43) and Kawthoung (*n* = 52). A summary of the demographic details of the patients who contributed samples to these three sites (with some overlap from the CQ efficacy study) is presented in Additional file 2. The isolates from Hpa-an (*n* = 10) and Insein (*n* = 1) were only included in country-wide population genetic summaries. A total of 52 and 22 of the day 0 samples from patients enrolled in the Kawthoung (87%) and Myawaddy (88%) clinical surveys were confirmed *P. vivax*-positive by PCR, with the remaining isolates failing to amplify any *Plasmodium* species.

#### Pvmdr1 copy number

The Thai breakpoint-specific *pvmdr1* amplification assay was applied to all 152 PCR-positive *P. vivax* isolates, with confident results (positive amplification of both test assays) in 86% of the isolates. As summarized in Table 2, a total of 2 (1.6%) isolates, including one from Shewgyin and one from Myawaddy, were positive for the *pvmdr1* amplification. Genotyping analysis revealed that both isolates comprised polyclonal infections (MOI = 2 and 3), and exhibited different multi-locus genotype profiles from one another (Table 3).

**Table 2:** Frequency of isolates with the Thai *pvmdr1* amplification-specific breakpoint in Myanmar

Site	No. successful assays (%)	% *pvmdr1* CN2+ isolates
Shwegyin	40/46 (87)	2.5% (1/40)
Myawaddy	31/43 (72)	3.2% (1/31)
Kawthoung	49/52 (94)	0% (0/49)
All Myanmar sites^a^	129/152 (85)	1.6% (2/129)

^a^Includes Shwegyin, Myawaddy, Kawthoung, Hpa-An, and Insein

**Table 3:** Within-host and population diversity

Site	No. successfully typed isolates (%)	% polyclonal infections	Mean MOI (range)	Mean *H*_E_ ± SD: all nine markers	Mean *H*_E_ ± SD: five balanced markers^a^
Shwegyin	46/46 (100)	33% (15/46)	1.41 (1–4)	0.876 ± 0.080	0.858 ± 0.061
Myawaddy	39/43 (91)	33% (13/39)	1.33 (1–2)	0.855 ± 0.065	0.849 ± 0.063
Kawthoung	47/52 (90)	36% (17/47)	1.64 (1–5)	0.861 ± 0.097	0.857 ± 0.057
All sites^b^	142/152 (93)	34% (48/142)	1.47 (1–5)	0.876 ± 0.076	0.859 ± 0.060

^a^MS1, MS5, MS10, MS12, MS20

^b^Includes Shwegyin, Myawaddy, Kawthoung, Hpa-An, and Insein

#### Population diversity and structure

##### Genotyping efficacy

A total of 142 (93%) isolates exhibited successful genotype calls at more than 50% of the nine loci assessed and were included in further population genetic analyses (Table 3). A detailed outline of the allele calls at each of the nine markers for each of the successfully genotyped samples is presented in Additional file 3. The three major sample groups each maintained sample size greater than 30 enabling confident population-level analyses; Shwegyin (*n* = 46), Myawaddy (*n* = 39) and Kawthoung (*n* = 47). All nine markers exhibited genotyping success rates >10% and displayed modest to high genetic diversity in each of the three major population groups, ranging from *H*_E_ = 0.667 (msp1F3 in Kawthoung) to 0.970 (MS16 in Shwegyin) (Additional file 4). All nine markers were, therefore, maintained in all subsequent population genetic analyses.

##### Temporal assessment of Shwegyin and Myawaddy

Parasite isolates from Shwegyin and Myawaddy were collected across multiple years, hence the analysis of parasite differentiation was undertaken in each study site after stratifying by year. There was no significant difference in the percentage of polyclonal infections, MOI, or expected heterozygosity (*H*_E_) in the Shwegyin parasites between 2012 and 2013 or between the Myawaddy parasites between 2012 and 2014 (all *P* > 0.05) (Additional file 5). Furthermore, the *F*_ST_ between the Bago 2012 and Bago 2013, and between the Myawaddy 2012 and Myawaddy 2014 samples was less than 0.05 indicative of little/no differentiation (Additional file 5). The samples were therefore pooled across years within each district to enhance the sample size.

##### Within-host and population diversity

**Fig. 3:**
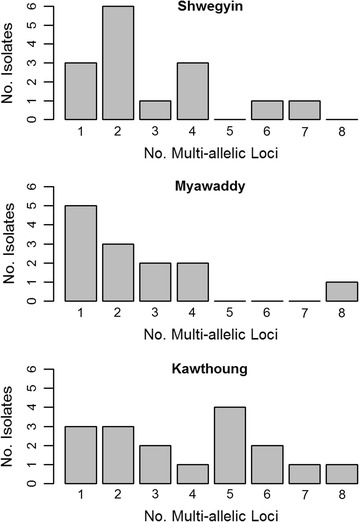
Barplots illustrating the frequency of multi-allelic loci in the polyclonal infections

Table 3 provides a summary of within-host and population-level diversity in each of the major study sites. Expected heterozygosity was high in each of Shwegyin (*H*_E_ = 0.876), Myawaddy (*H*_E_ = 0.855) and Kawthoung (*H*_E_ = 0.861). When analysis was restricted to the five balanced markers, expected heterozygosity estimates were slightly lower than with the full set of nine markers, but remained high in each population (mean *H*_E_ range 0.849–0.858). Rates of polyclonal infection and mean multiplicity of infection (MOI) were similar among the three sites, ranging from 33–36% to 1.33–1.64 respectively. A large proportion of the polyclonal infections exhibited multiple alleles at more than one locus (range 2–8 multi-allelic loci), accounting for 80% of the polyclonal infections in Shwegyin, 62% in Myawaddy and 82% in Kawthoung. As illustrated in Fig. 3, the polyclonal isolates in Kawthoung exhibited higher levels of within-host diversity than the other sites, with the most common number of multi-allelic loci = 5.

##### Population structure and differentiation

According to the definitions of Balloux et al. the differentiation between the three major sites was moderate when the classic *F*_ST_ metric was applied (*F*_ST_ range 0.016–0.026, all *P* < 0.05), and moderate to high when the standardized (*F*_ST_^′^) metric was applied (*F*_ST_^′^ range 0.117–0.191, all *P* < 0.05) (Table 4). However, when the five balanced markers were applied, the differentiation in all pairwise comparisons was low with the classic *F*_ST_ metric (*F*_ST_ range 0.003–0.009, all *P* > 0.05), and low to moderate when the *F*_ST_^′^ metric was applied (*F*_ST_^′^ range 0.019–0.065, all *P* > 0.05). STRUCTURE analysis also failed to identify any evidence of sub-structure within the population. Although the delta *K* method is not able to assess the probability of *K* = 1 (i.e. one population with no sub-structure), the highest delta *K* value for the remaining *K* estimates was very low, with a score of 10.6 at *K* = 2 (Additional file 6). Inspection of the bar plots at *K* = 2 revealed that all isolates were equal mixtures of two populations indicating that STRUCTURE was unable to separate them into two distinct sub-populations.

**Table 4:** Pair-wise differentiation between sites

Markers	Sites	Shwegyin	Myawaddy	Kawthoung
Nine markers	Shwegyin	–	0.191	0.117
	Myawaddy	0.026 (*P* = 0.000)	–	0.161
	Kawthoung	0.016 (*P* = 0.011)	0.022 (*P* = 0.003)	–
Five balanced markers^a^	Shwegyin	–	0.055	0.065
	Myawaddy	0.008 (*P* = 0.129)	–	0.019
	Kawthoung	0.009 (*P* = 0.109)	0.003 (*P* = 0.322)	–

*F*_ST_ (*P value*) in lower left triangle. *F*_ST_^′^ in upper right triangle

^a^MS1, MS5, MS10, MS12, MS20

##### Relatedness

**Fig. 4:**
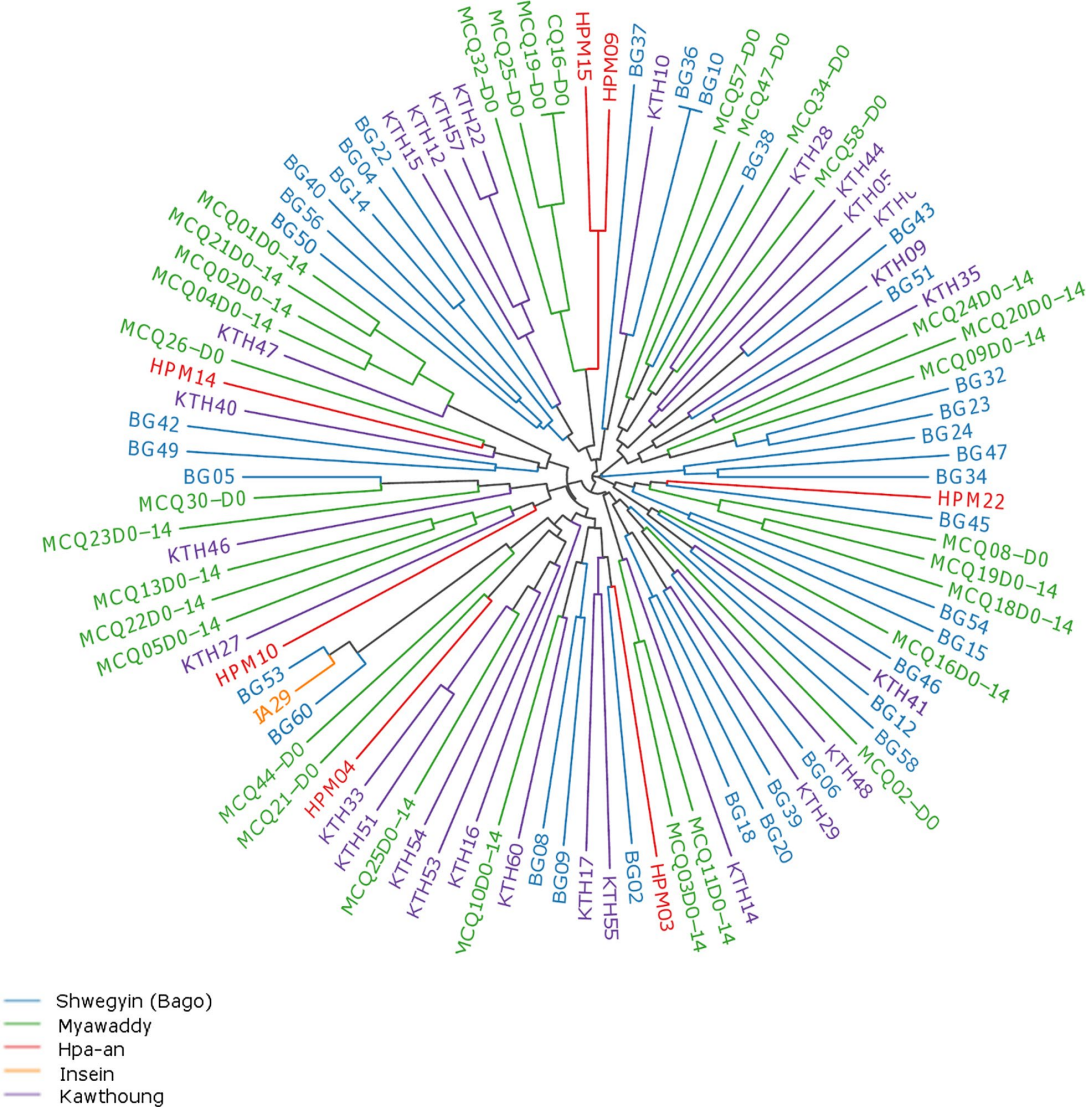
Unrooted neighbour-joining tree illustrating the genetic relatedness between the *P. vivax* isolates within and among different districts in Myanmar. Only the isolates with complete allelic data across all nine loci are included in the analysis (*n* = 103)

Neighbour-joining analysis highlighted the extensive population-level diversity and low relatedness between the large majority of *P. vivax* isolates in all three populations (Fig. 4). Only two pairs of isolates (one pair from Shwegyin and one pair from Myawaddy) exhibited identical MLGs. As observed with STRUCTURE analysis, neighbour-joining analysis did not reveal any notable clustering reflective of sub-populations within the dataset.

##### Linkage disequilibrium

When all isolates with complete data across all nine loci were assessed, LD analysis revealed significant but moderately low LD in Shwegyin (*I*_A_^S^ = 0.023, *P* = 0.01), Myawaddy (*I*_A_^S^ = 0.071, *P* = 0.01) and Kawthoung (*I*_A_^S^ = 0.045, *P* = 0.01) (Table 5). Similar results were observed when analysis was restricted to the low complexity samples inferring that the results in all isolates (and other MLG-based analyses in the study) did not appear to be affected by potential artificial recombinants in polyclonal infections (Table 5). As very few MLGs were present in more than one isolate, when the dataset was restricted to unique MLGs, the LD results were very similar to the results observed in all isolates, with no evidence of epidemic transmission dynamics (Table 5).

**Table 5:** Linkage disequilibrium

Site	All infections		Low complexity^a^		Unique MLGs	
	*N*	*I*_A_^S^	*N*	*I*_A_^S^	*N*	*I*_A_^S^
Shwegyin	35	0.023*	25	0.020*	34	0.010^NS^
Myawaddy	33	0.071*	26	0.105*	31	0.059*
Kawthoung	27	0.045*	19	0.021*	27	0.045*
Myanmar	103	0.015*	75	0.020*	101	0.012*

Only samples with no missing data at all nine loci are included in the analyses

^NS^ Not significant (*P* > 0.05), * *P* < 0.05, ** *P* < 0.01, *** *P* < 0.001

^a^Restricted MLGs from samples with no more than one multi-allelic locus

## Discussion

This study used a combination of clinical and molecular tools to gain insight into chloroquine efficacy, the pressure of ‘second-hand’ exposure to ASMQ, and local patterns of diversity, structure and associated transmission intensity and stability in the *P. vivax* population in south-eastern Myanmar. The three cases of treatment failure following CQ monotherapy may reflect under-dosing with CQ or demonstrates low level evidence of chloroquine resistance. Molecular assessment of *pvmdr1* CN amplification infers very low pressure from ASMQ, with patterns of population diversity and structure which are consistent with moderately stable transmission, and early evidence of declining transmission.

The clinical efficacy study suggests reasonable overall efficacy levels of CQ, with chloroquine efficacy greater than 90% in both sites. Results from the site in southern Myanmar (Kawthoung) showed high CQ efficacy, whereas in Myawaddy the risk of recurrence was 8%. The mean CQ dose was lower in patients with recurrent parasitaemia, suggesting that suboptimal dosage might have contributed to this finding other work of pooled data across several studies suggests similar findings [38]. The results could also be caused by poor drug absorption or reduced drug quality. Although analysis of chloroquine blood concentrations and drug quality would help to clarify this and confirm true chloroquine resistance, these were not collected in this study.

In *P. falciparum* infection, parasite recrudescence is associated with higher parasitaemia at the start of treatment [39, 40], and this is associated with prolonged clearance. Delayed parasite clearance is a known risk factor for treatment failure of *P. vivax* and proposed as a useful surrogate marker of chloroquine sensitivity [5, 41]. Parasite clearance was actually not significantly faster in Myawaddy, despite higher baseline parasitaemia suggesting that factors other than resistance may have influenced the lower efficacy estimates.

Reduced CQ sensitivity is reported from north-eastern Myanmar [9] where CQ, despite being co-administered with 8 days of primaquine (PQ), resulted in only 94.8% efficacy. In the south of the country a larger study of 250 patients found more than 30% of patients treated with CQ as certainly or probably resistant [7]. However CQ appears to retain good efficacy along the China–Myanmar border [42] and in other sites [10]. These findings indicate significant heterogeneity across the country warranting more focal drug resistance monitoring.

Molecular surveillance of the *pvmdr1* CN amplification observed commonly in the Thai border region with Myanmar demonstrated that the amplification was present at very low frequency in Myawaddy (3.2%) and Shwegyin (2.5%). These results concur with a previous study which used real-time PCR to quantify *pvmdr1* CN, identifying CN amplifications in just 2% (1/49) of isolates from Myanmar in 2008 [13]. The survey by Imwong and colleagues found the same genetic background in *pvmdr1* CN amplifications from Myanmar, Laos and Thailand, suggesting a common origin, most likely from Thailand, where MQ pressure has historically been greatest and *pmvdr1* CN accordingly most prevalent [13, 43]. The *pvmdr1* CN amplifications observed in this study most likely also originated from Thailand, but remained at low frequency owing to low levels of MQ pressure on the *P. vivax* population. The low MQ pressure may reflect that ASMQ is currently used as second-line therapy for *P. falciparum* in Myanmar—for treatment of infections that have failed to clear after an initial course of AL. It should be noted that if *pvmdr1* CN amplifications with differing breakpoints than those observed in Thailand are present in south-eastern Myanmar, it is possible that the rate of amplification and associated ASMQ pressure is higher than estimated here. Further monitoring is required of both MQ pressure and, once suitable markers are available for *P. vivax*, of artemisinin and other partner drugs.

In the current study, all three sites demonstrated high population diversity (*H*_E_ range 0.85–0.88). Comparably high levels of diversity have been observed in the majority of *P. vivax* studies to date, across a wide range of endemicities [44], with the exception of a few pre-elimination settings in Malaysia [45], Peru [46] and the Republic of Korea [47], where lower *H*_E_ ranging from 0.4 to 0.7 were observed. In some studies, a correlation between diversity and endemicity may have been obscured by markers with excess diversity [28]. However, even after restricting analysis to five markers with balanced diversity (MS1, MS5, MS10, MS12 and MS20), *H*_E_ levels remained high in Myanmar (*H*_E_ range 0.85–0.86), indicative of high transmission.

Previous microsatellite-based studies conducted on isolates collected from across Kayin, Kachin and Rakhine States in 2007, and on isolates collected from Kachin State between 2011 and 2013 demonstrated equivalently high diversity (*H*_E_ range 0.82–0.85) [48, 49], although direct comparisons are complicated by differences in the marker panels between the studies. The 2007 study reported 67% polyclonal infections across the three states investigated, in contrast to just 29% in the Kachin study, and 34% (range 33–36%) in the current study. This difference may reflect changes in local *P. vivax* transmission between 2007 and 2012–14, but comparisons are again limited. More effective comparisons are possible with other studies that have applied the APMEN markers, collectively revealing a spectrum of polyclonality rates across a range of endemic settings; 3% in the Republic of Korea [47], 4–12% in Central China [50], 26% in Malaysia [45], 30% in the Solomon Islands [51], 8–67% in Ethiopia [52], 65% in Iran (including imported infections from Afghanistan and Pakistan) [25], and 23–70% in Indonesia [53]. In this context, the rates of polyclonal infection in Myanmar were most comparable to low endemic settings in the tropics. However, in contrast to the low endemic settings in Malaysia, the Solomon Islands and Indonesia, the majority of polyclonal infections in Kawthoung and Shwegyin were multi-allelic at multiple loci, indicative of highly complex infections. This paradox of moderately low proportions of polyclonal infection, with high infection complexity might reflect local heterogeneity in transmission such as ‘hot-spots’ or ‘hot-pops’. Further investigation to identify any epidemiological links between the highly complex infections would provide insights that might be useful for identifying target areas for resource prioritization.

Neighbour-joining analysis illustrated the high population diversity, and low relatedness between infections other than a few clusters in Myawaddy. STRUCTURE analysis also failed to identify any evidence of sub-structure within Myanmar. Furthermore, LD was moderately low in all three sites (*I*_A_^S^ range 0.023–0.071, all *P* < 0.05), suggestive of relatively high rates of recombination. In accordance with the modest clustering by neighbour-joining analysis, and comparatively lower polyclonal infection complexity relative to Shwegyin and Kawthoung, the highest level of LD was observed in Myawaddy (*I*_A_^S^ = 0.071). Collectively, these findings infer moderately stable *P. vivax* transmission, with perhaps the first hints of interruption to transmission in Myawaddy. In accordance with the NMCP category 1 API classifications, the genotyping results support ongoing *P. vivax* transmission reduction (control) activities in all three sites. The genetic data presented in this study provides a useful baseline for the population genetic analysis of the impact of ongoing public health interventions to reduce transmission, to which contemporary data can be added.

The available data suggests that Myawaddy has made the greatest progress in reducing local *P. vivax* transmission, but diligent surveillance is essential to avoid re-introducing cases from Shwegyin, Kawthoung and other sites. Indeed, *F*_ST_ analysis revealed low differentiation between the sites, particularly when the 5 balanced markers were implemented (*F*_ST_ range 0.003–0.009, all *P* > 0.05; *F*_ST_^′^ range 0.019–0.065, all *P* > 0.05).

## Conclusions

Parasite recurrence was observed in Myawaddy and Kawthoung, along with evidence of *pvmdr1* amplification emphasizes the importance of ongoing drug resistance surveillance in the *P. vivax* population in Myanmar. This is further underscored by the genotyping data, which found no evidence of physical, social or other boundaries that would impede the spread of resistant infections between sites.

## Additional files

**Additional file 1.** Site details.

**Additional file 2.** Patient demographic details for the successfully genotyped samples from Shwegyin, Kawthoung and Myawaddy.

**Additional file 3.** Genotype calls at 9 markers in 142 successfully genotyped *P. vivax* PCR-positive isolates.

**Additional file 4.** Marker diversity as measured by the expected heterozygosity.

**Additional file 5.** Within-host and population diversity by year in Shwegyin and Myawaddy.

**Additional file 6.** Delta K assessment of STRUCTURE output on 142 *P. vivax* isolates from Myanmar.
