# Supplementary material for: Chloroquine efficacy for Plasmodium vivax in Myanmar in populations with high genetic diversity and moderate parasite gene flow
Source: Malar J. 2017 Jul 10;16:281. doi: 10.1186/s12936-017-1912-y (PMC5504659; doi:10.1186/s12936-017-1912-y)
Supplement: Supplementary file 1 — Additional file 1. Site details. [file 12936_2017_1912_MOESM1_ESM.docx]

**Table S1. Site details**

| **Site** | **State/Division** | **Enrolment center** | **Enrolment framework** | **Enrolment period** | **Division level all malaria API in 2012** ^1^ | **Division level *P. vivax* API in 2012** ^1,2^ |
| --- | --- | --- | --- | --- | --- | --- |
| Shwegyin | Bago Division | Sub-centre of RHC at Taung Pat and Kun Seik villages | Cross-sectional survey | September 2012 - June 2013 (10 months) | 4.85 | 1.36 |
| Myawaddy | Kayin State | Pwint Lin Aye Myaing village | Cross-sectional survey in 2012; CQ efficacy survey in 2014 | September 2012 – November 2012 (3 months); August 2014 –September 2014 (2 months) | 9.70 | 2.72 |
| Kawthoung | Tanintharyi Division | Yuna Palm Oil project hospital | Cross-sectional survey | August 2012 – November 2012 (4 months) | 21.67 | 6.07 |
| Hpa-an | Kayin State | Hpa-an Hospital | Cross-sectional survey | May 2014 – December 2014 (8 months) | 9.70 | 2.72 |
| Insein | Yangon Division | Insein Hospital | Cross-sectional survey | July 2014 – December 2014 (6 months) | 0.25 | 0.07 |

^1^ Annual parasite incidence (API) expressed as cases per 1000 population of the given state/division in 2012. ^2^ Based on an estimated 0.28% *P. vivax* cases in 2012. Details on the API and percentage of *P. vivax* cases were provided by Dr Katherine Battle, Malaria Atlas Project.
