# Supplementary material for: Chloroquine efficacy for Plasmodium vivax in Myanmar in populations with high genetic diversity and moderate parasite gene flow
Source: Malar J. 2017 Jul 10;16:281. doi: 10.1186/s12936-017-1912-y (PMC5504659; doi:10.1186/s12936-017-1912-y)
Supplement: Supplementary file 3 — Additional file 3. Genotype calls at 9 markers in 142 successfully genotyped P. vivax PCR-positive isolates. [file 12936_2017_1912_MOESM3_ESM.docx]

**Table S2. Patient demographic details for the successfully genotyped samples from Shwegyin, Kawthoung and Myawaddy.**

| **Site** | **Collection period** | **Median patient age, years (range)** | **% Male patients (No. males/ No. females)** | **Median parasite density, parasites per microliter (range)** |
| --- | --- | --- | --- | --- |
| Shwegyin | Jun 2012 – Sep 2013 | 21 (6-50) | 80% (37/46) | 4,267 (284 – 26,399) |
| Myawaddy | Sep 2012 – Nov2012; Aug 2012 – Sep 2014 | 22 (6 – 48) | 82% (32/39) | 9,940 (403 – 38,970) |
| Kawthoung | Aug 2012 – Nov 2012 | 24 (6 - 56) | 66% (31/47) | 6,868 (173 – 66,220) |
