# Supplementary material for: Chloroquine efficacy for Plasmodium vivax in Myanmar in populations with high genetic diversity and moderate parasite gene flow
Source: Malar J. 2017 Jul 10;16:281. doi: 10.1186/s12936-017-1912-y (PMC5504659; doi:10.1186/s12936-017-1912-y)
Supplement: Supplementary file 4 — Additional file 4. Marker diversity as measured by the expected heterozygosity. [file 12936_2017_1912_MOESM4_ESM.docx]

**Table S4. Marker diversity as measured by the Expected Heterozygosity**

| **Marker** | **Shwegyin** | **Kawthoung** | **Myawaddy** | **All Myanmar** |
| --- | --- | --- | --- | --- |
| MS8 | 0.960 | 0.943 | 0.949 | 0.953 |
| MS16 | 0.970 | 0.960 | 0.783 | 0.947 |
| pv3.27 | 0.920 | 0.941 | 0.902 | 0.941 |
| MS10 | 0.948 | 0.905 | 0.920 | 0.926 |
| MS20 | 0.854 | 0.906 | 0.889 | 0.888 |
| MS5 | 0.883 | 0.838 | 0.862 | 0.880 |
| MS12 | 0.808 | 0.821 | 0.817 | 0.815 |
| MS1 | 0.796 | 0.766 | 0.758 | 0.776 |
| msp1f3 | 0.745 | 0.667 | 0.815 | 0.757 |
| Mean | 0.876 | 0.861 | 0.855 | 0.876 |
