# Supplementary material for: Chloroquine efficacy for Plasmodium vivax in Myanmar in populations with high genetic diversity and moderate parasite gene flow
Source: Malar J. 2017 Jul 10;16:281. doi: 10.1186/s12936-017-1912-y (PMC5504659; doi:10.1186/s12936-017-1912-y)
Supplement: Supplementary file 5 — Additional file 5. Within-host and population diversity by year in Shwegyin and Myawaddy. [file 12936_2017_1912_MOESM5_ESM.docx]

**Additional File 5. Within-host and population diversity by year in Shwegyin and Myawaddy**

| **Site/Year** | **No. successfully typed isolates (%)** | **% Polyclonal infections** | **Mean MOI (range)** | **Mean *H*_E_ ± SD: all 9 markers** | **Mean *H*_E_ ± SD: 5 balanced markers ^1^** | ***F*_ST_: 9 markers** | ***F*_ST_: 5 balanced markers ^1^** |
| --- | --- | --- | --- | --- | --- | --- | --- |
| Shwegyin 2012 | 18/18 (100%) | 44% (8/18) | 1.611 (1-4) | 0.864 ± 0.088 | 0.835 ± 0.091 | - | - |
| Shwegyin 2013 | 28/28 (100%) | 25% (7/28) | 1.286 (1-3) | 0.881 ± 0.077 | 0.875 ± 0.055 | - | - |
| **Shwegyin 2012 vs 2013** | **18 vs 28** | ***P*= 0.293** | ***P*= 0.155** | ***P*= 0.596** | ***P*= 0.691** | **0.017 (*P*= 0.080)** | **0.013 (*P*= 0.198)** |
| Myawaddy 2012 | 18/19 (95%) | 33% (6/18) | 1.333 (1-2) | 0.844 ± 0.094 | 0.806 ± 0.115 | - | - |
| Myawaddy 2014 | 21/24 (88%) | 33% (7/21) | 1.333 (1-2) | 0.827 ± 0.076 | 0.841 ± 0.060 | - | - |
| **Myawaddy 2012 vs 2014** | **18 vs 21** | ***P*= 0.733** | ***P*= 1.00** | ***P*= 0.547** | ***P*= 0.690** | **0.035 (*P*= 0.020)** | **0.049 (*P*= 0.008)** |

^1^ MS1, MS5, MS10, MS12, MS20
