# Supplementary figures and images for: Chloroquine efficacy for Plasmodium vivax in Myanmar in populations with high genetic diversity and moderate parasite gene flow
Source: Malar J. 2017 Jul 10;16:281. doi: 10.1186/s12936-017-1912-y (PMC5504659; doi:10.1186/s12936-017-1912-y)

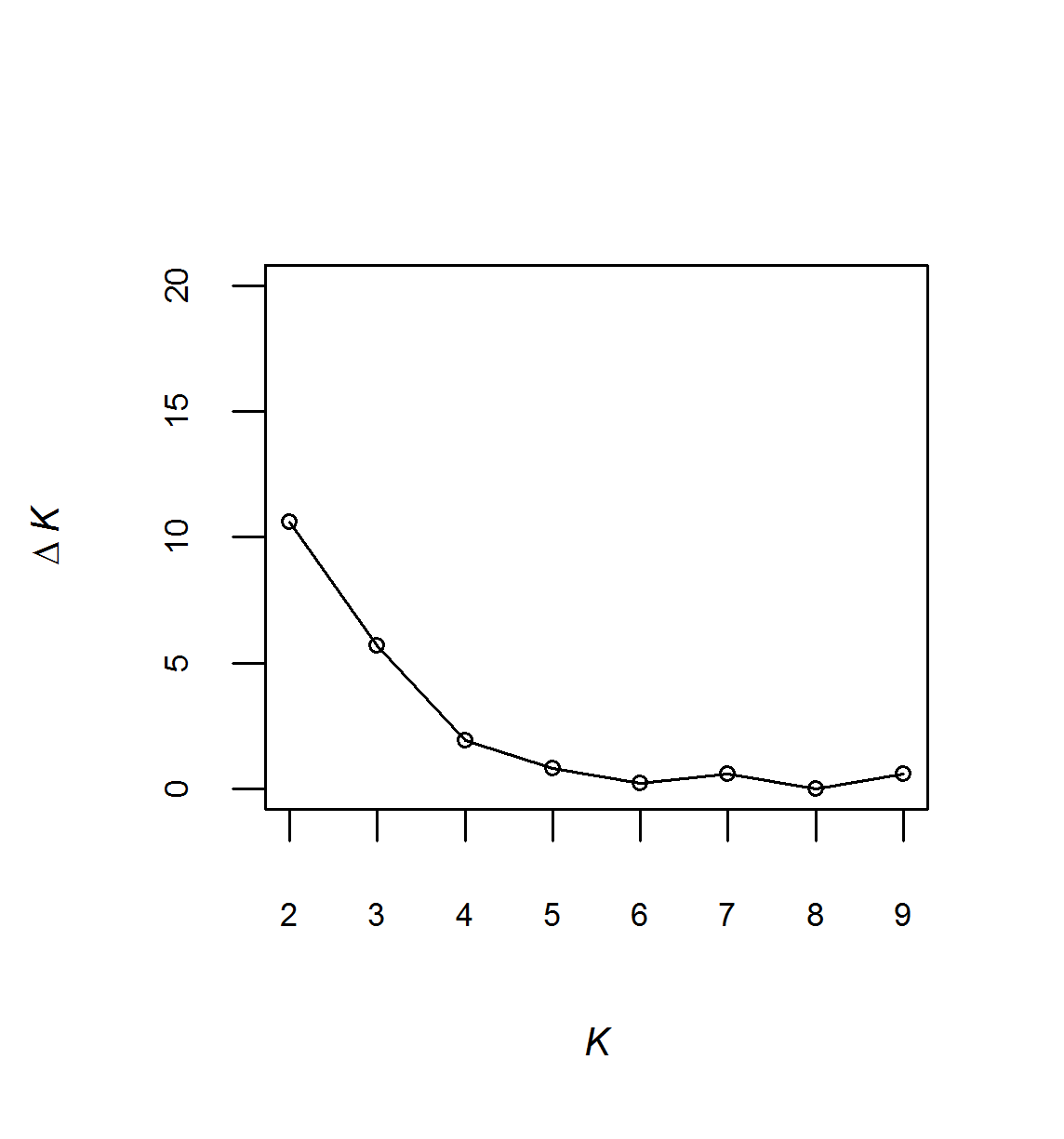

Supplement: Supplementary file 6 — Additional file 6. Delta K assessment of STRUCTURE output on 142 P. vivax isolates from Myanmar. [file 12936_2017_1912_MOESM6_ESM.tiff]
